# Payment models and the sustainability of community pharmacy practice: a qualitative interview study with community pharmacists

**DOI:** 10.1080/20523211.2025.2450018

**Published:** 2025-01-13

**Authors:** Kelly Ann Schmidtke, Terri Warholak

**Affiliations:** a Liberal Arts Department, University of Health Sciences and Pharmacy, St Louis, MO, USA; b College of Pharmacy, University of Health Sciences and Pharmacy, St Louis, MO, USA

**Keywords:** Community pharmacy services, health policy, reimbursement mechanisms, organisational policy

## Abstract

**Background:**

The sustainability of community pharmacies in the United States depends, in large part, on policies enacted by the Centers for Medicare and Medicaid Services (CMS). In 2003, CMS policy allowed retrospective direct and indirect remuneration (DIR) fees to manage costs. From 2024, only prospective DIR fees are permitted. The current study explores how existing payment models have impacted practice and how the policy change might impact future practice.

**Methods and Materials:**

Semi-structured qualitative interviews were conducted with community pharmacists knowledgeable about third-party payment and reimbursement practices in the state of Missouri. Interviews were recorded, transcribed, and reflectively analysed to identify broad themes. Final codes were applied to direct quotes. Participants checked transcripts and drafts of the manuscript for accuracy and completeness.

**Results:**

Twelve pharmacists (11 males) with self-expressed knowledge of fees impacting their practice(s) participated. The pharmacists owned or worked for community pharmacies. The median percentage of patients served on Medicare Part D was 35% (range 24% to 60%). Four main themes and one overarching theme were identified. Theme 1 describes a sense of being punished for the basic component of pharmacy practice, i.e. safely dispensing prescription medications. Theme 2 describes a diversification of the business model to subsidise losses on the basic component. Theme 3 describes anticipated challenges given the policy change. Theme 4 describes what may be needed to achieve payment reform. The overarching theme describes the purpose of community pharmacy, including who community pharmacists are and who they serve, i.e. their community.

**Conclusion:**

Community pharmacies require a financially viable and sustainable business model to deliver the legally required basic component of practice: safely dispensing prescription medications. Legislative action could help to ensure community pharmacies are appropriately compensated for work at the federal and state levels. Where services beyond the basics cost-effectively benefit public health, payment models could support them.

## Background

Community pharmacies offer services to improve public health in the places people live (Valliant et al., 2022). Continuing to serve the community requires a sustainable business model that supports the basic component of pharmacy practice: safely dispensing and/or administering prescription medications and immunizations in compliance with all laws and practice standards (Rupp, 2020). In alignment with the Omnibus Budget Reconciliation Act in the United States, safely dispensing medications requires appropriate compensation for medications and operating costs, including costs for conducting standard drug utilisation reviews and patient counselling (United States Congress, 1990). Without appropriate compensation, this basic component of community pharmacy practice is increasingly unsustainable, and the more aspirational goals of pharmacy are less likely to be realised, e.g. improving patient outcomes.

The largest single-payer of prescription medications in the United States is the Centers for Medicare and Medicaid Services (CMS, 2023). As of 2023, approximately fifty million Americans are enrolled in the Medicare Part D programme (CMS, n.d.). To pay for medications, CMS contracts with private insurers (e.g. UnitedHealthcare, Aetna), which contract through pharmacy benefit managers (e.g. CVS Caremark, Express Scripts, Optum) to negotiate rates and process claims. Before managed care, pharmacy practice was largely reimbursed based on charges that were usual, customary, and reasonable. Such reimbursements could involve discounts that increased profits earned on the medications. When managed care was introduced, pharmacy benefit managers created a two-component reimbursement model, which included a discount off the published index price of the medication and a flat dispensing fee intended to reflect operating costs. While the set dispensing fee was too low to cover many practices’ operating costs, this model was accepted by many pharmacy owners because they were routinely purchasing medications for a greater discount than the formula was paying (Rupp, 2020).

To avoid overspending, the Medication Modernization Act required pharmacy benefit managers to report all discounts given after the drug was sold to CMS (United States Congress, 2003). From there, reimbursement to pharmacies involved at least two steps. First, pharmacy benefit managers reimbursed pharmacies at an anticipated amount as soon as possible. Second, because some discounts could not be known before dispensing, pharmacy benefit managers requested additional money three to six months later. The money requested later came to be called retrospective direct and indirect remuneration (DIR) fees (Frier Levitt LLC, 2017)

Initially DIR fees were low, but they gradually increased. CMS’s 2014 Final Rule redefined ‘negotiated prices' to exclude contingent pharmacy payment adjustments, which legally permitted pharmacy benefit managers to increase retrospective fees (United States Department of Health and Human Services, 2014). The largest increases coincided with an expanded use of performance metrics, many of which are indirect measures of patient adherence. In 2022, CMS’s Final Rule revised the definition of ‘negotiated price' to eliminate retrospective DIR fees (CMS, 2022). From January 2024, fees must proactively reflect the price consumers pay at the point of sale at a pharmacy. This policy change does not eliminate fees.

Policy papers, commentaries, and editorials discuss the impact of increasing DIR fees (e.g. see Gabay, 2017) and other hidden practices (e.g. Mattingly et al., 2023) but lack comprehensive descriptions of how these routines influence pharmacy practice. The Academy of Managed Care Pharmacy (2022) has pushed for greater transparency in reimbursement rates. However, transparency alone may not improve sustainability where it only reveals unsustainable losses. In Urick et al.’s 2021 online survey, most pharmacy owners (90%, N = 27) endorsed items stating that they had lost money because of performance-based metrics and that actions necessary to avoid penalties were beyond their control. Despite these reactions, most owners reported expanding services. It is unclear how such expansions could impact the sustainability of community pharmacy.

Richard et al.’s (2021) large mixed-methods study identified contextual and motivational influences needed for successful implementation of performance-based pharmacy payment models. They found that successful implementation requires alignment of stakeholder aims, cultural shifts that support quality improvement, and targeted financial incentives for specific pharmacies rather than plan-base groups. Whether or not these influencers have been realised to improve the implementation of such models in pharmacy is an open question. The present study provides some answers.

The present study aims to explore the impact of existing payment models on community pharmacy. The interviews were conducted two to three months before the DIR policy change. These qualitative data allow insights into how community pharmacists perceive existing fees shaped practice and how the policy change might impact future practice.

## Method

### Ethical approval

The University of Health Sciences and Pharmacy ethically approved the study (IRB Code: 2023-12). All participants provided informed consent via an emailed online survey before participating. The information sheet explained that the purpose of the study was to learn about how fees were impacting their practice, that participation was voluntary, and that identifiable data would be redacted from reports. The study is reported in accordance with the consolidated criteria for reporting qualitative research checklist (COREQ; see Supplemental Appendix 1; Tong et al., 2007).

### Study design

Semi-structured qualitative interviews were conducted. We chose a qualitative method to capture more nuanced impressions of how DIR fees influenced pharmacy practice and might influence future practice. The methodological approach was pragmatic. No prior framework or theory was assumed before data collection commenced (Ramanadhan et al., 2021). The semi-structured interview guide (see Supplemental Appendix 2) was co-created by the primary investigator (KAS) and a pharmacist who had over 30 years of experience (TW; RPh, FAPhA; PhD; Female). The guide was piloted with two professors of pharmacy practice (1 male and 1 female) before recruitment commenced.

### Sample size/participant selection

Our sample size was informed by the idea of information power, such that only a small set of participants very knowledgeable about their practice were needed to identify broad themes (Malterud et al., 2016). Participation was limited to pharmacists in the state of Missouri who were knowledgeable about fees impacting their practice. Participants had no prior relationship with the interviewer. Initial participants were purposively identified through local networks. Additional participants were recruited via snowball methods. Five professionals we contacted stated reasons for not participating including having retired, not having time, and not knowing about their DIR fees.

### Data collection

Participants completed an online survey about themselves and their pharmacies (location, number of stores, number of employees, percent of patients who were Medicare Part D beneficiaries, and estimated annual DIR fees for 2022 and 2023). Then, participants scheduled an interview with an assistant professor of psychology who had training in qualitative research and over ten years of experience working in applied healthcare (KS, PhD, Female). Interviews were conducted in September and October 2023 and were recorded via Microsoft Teams or telephone. Participants took part in a location of their choosing and the interviews lasted approximately 30 minutes (Median = 26 minutes, range 21 to 58 minutes). The interviewer took field notes during each interview and then wrote a summary encapsulating the main points after the interview. During the transcription of the interviews, the research team redacted identifiable information. Deidentified transcripts were sent to participants. Participants were given two weeks to make revisions before our analyses commenced. Four participants made revisions that involved correcting spellings for medication names or organisation names and defining previously undefined acronyms. No participants withdrew after reviewing their transcripts.

### Data analysis

Transcripts were analysed using Braun and Clarke’s (2006) thematic method. Five members of the research team participated in this iterative and reflective process, including the interviewer (KS), the previously mentioned pharmacist (TW), and three students currently enrolled in pharmaceutical studies (ZV, VS, and YJ). Initial codes were created and revised to identify coherent themes with nine transcripts. A concept map was created in PowerPoint with hierarchical levels. Based on consensus discussions, a final coding guide was developed. Then, three additional transcripts were reviewed by the research team who agreed that thematic saturation was achieved (Saunders et al., 2018). A copy of the final report was sent to participants, who were offered the opportunity to express their questions, comments, and concerns before the manuscript was submitted for publication.

## Results

### Participant demographics / pharmacy characteristics

Table 1 describes the participant demographics and pharmacy characteristics. Twelve participants (11 male) took part. One participant identified as a business consultant, two as presidents, two as the pharmacist in charge, and seven as owners. Participants worked with their current practice for six or more years. Ten participants worked with independent pharmacies, defined as having three or fewer stores. One participant worked with 5 stores, and the business consultant worked with 18 stores within Missouri and 50 in other states. Seven participants’ stores were located in urban areas, three in rural areas, and two in a mix. The median percentage of patients served on Medicare Part D was 35% (range = 24% to 60%). All but one participant anticipated an increase in DIR fees from 2022 to 2023; the median increase was 13% (range = −13% to 25%).

**Table 1.** Participant demographics and pharmacy characteristics.

Participant ID/Gender	Title	Years working	Location (urban or rural)	Number of stores in Missouri	Number of full-time staff employed	What percent of business is Medicare Part D?
P1/Male	President	>20	Urban	2	>20	51%
P2/Male	President	>20	Urban	2	10	60%
P3/Male	Owner	14	Urban	2	18	32%
P4/Male	Owner	12	Urban	1	<10	30%
P5/Male	Pharmacist in Charge	>20	Urban	2	<10	36%
P6/Male	Owner	>20	Rural	3	>20	33%
P7/Male	Owner	>20	Mix	5	<10	25%
P8/Male	Owner	15	Rural	3	20	40%
P9/Male	Pharmacist in Charge	>20	Rural	1	10	37%
P10/Male	Owner	>20	Urban	2	>20	35%
P11/Female	Owner	6	Urban	1	<10	24%
P12/Male	Pharmacist Business Consultant	>20	Mix	18^a^	>20	24%

^a^ an additional 50 pharmacies in Illinois

### Themes

**Figure 1.**
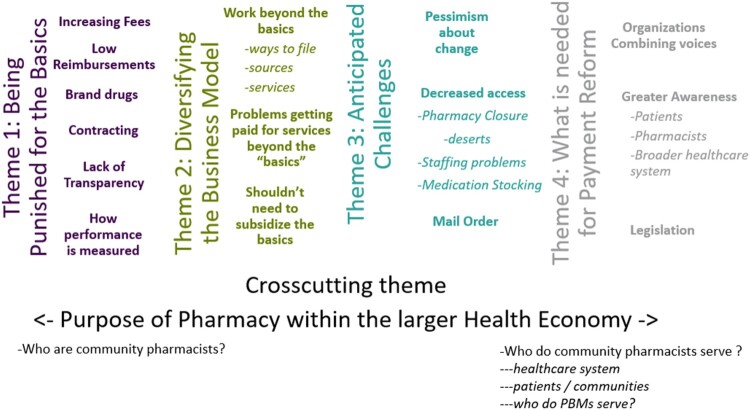
Concept map.

We identified four main themes and one overarching theme. The main themes are related to (1) feeling punished for the basic component of pharmacy practice, (2) diversifying the business model, (3) anticipating consequences of the new policy, and (4) identifying what is needed for payment reform. The overarching theme spoke to the purpose of community pharmacy. A concept map describing the themes and subtheme relationships is in Figure 1. A larger version of the concept map is in Supplemental Appendix 3 with additional quotes. The four main themes are described below. Quotes representing the overarching theme are interweaved into the main themes.

#### Theme 1: being punished for the basics

The first theme captures how pharmacists feel that they are being punished for safely dispensing medications. This relates to the purpose of community pharmacy (the overarching theme). Participant 3 stated, ‘Pharmacy’s sole purpose is for the safety of the patients. Yet, they can't regulate that when there's no money to pay people.' Some participants expressed concerns about not having the funds available to replace equipment or hire staff. Participant 5 reported, ‘We're being paid pennies to fill a prescription. In some cases, we're being paid negative pennies to fill prescriptions.'

All participants expressed concerns about how quickly the DIR fees increased and concerns that even top-performing pharmacies faced penalty fees. Several participants provided statistics about the increase over the previous decade, ranging from 91,500% to 110,000%. The increases affected both generic and brand-name medications, but greater concern was expressed about brand-name medications. Providing an example of negative reimbursement rates, Participant 10 said, ‘If the drug costs $500.00 for the brand, I'll get reimbursed, say, $550, and then my DIR fee is $60.00. So, I've just lost $10, and that is 100% true. Verifiable on most brand name drugs, we ultimately lose money on a Part D plan.'

Plan contracts could include the fee schedule and goalposts for performance, but the details are often obscure. As Participant 11 notes, ‘It’s the most zigzag line you could have. I think that's partly on purpose.' Participant 8 said, ‘What I've seen in most of the DIR contracts has been very skewed, contracts set up in a way that you pretty much could never win.' Further frustrations were expressed about the goalposts changing, sometimes within contracted periods. Others expressed frustration about how adherence was measured. For instance, Participant 1 argues that 100% compliance is not obtainable:

If anybody says that ‘you should have 100% compliance' I would refer them to fraud, waste, and abuse. If you have 100% compliance you have done one of three things, either: no one has ever died in your pharmacy, no one has ever gone to the hospital in your pharmacy, or no one has ever gone to rehab in your pharmacy. It means that every individual is taking every tablet, every day on time for the whole year.

Contracting itself was felt to be a time-consuming practice. To save time, many participants depended on Pharmacy Service Administrative Organizations (PSAOs) to negotiate contracts. In these cases, the DIR fees could be based on the performance of all pharmacies within a PSAO's chain code. Participant 6 explains, ‘The DIR calculation is based on something that is not, in many ways, it's not movable at the pharmacy level. … It's not a pharmacy measure. It's a health plan measure.'

Participants largely believed that the original purpose of DIR fees was to improve adherence. However, now, they question whether the existing payment models could incentivize improvement. Participant 10 reflected:

When you look at the money that we spend for it, doing adherence and all the other stuff, I paid over $500,000 in DIR fees. What am I going to earn back? $10,000, $20,000. It's insignificant. So, if I'm paying people to improve adherence, say I hire somebody and I'm paying them $50,000 a year, well, it probably doesn't even pay for that person.

#### Theme 2: diversifying the business model

To make up for money lost on the basic component, pharmacists are expanding more traditional services and developing new services. For instance, some participants discussed new procedures and practices that could promote medication adherence, e.g. medication delivery services or blister packaging. Participant 10 said they devote a lot of attention to the front of the store (e.g. where cosmetics and toiletries are sold), stressing, ‘Frankly it’s what allows me to continue to put food on the table.' Some participants filed paperwork to be reimbursed as long-term care pharmacies. However, opportunities for long-term care might be limited to cases where pharmacies serve eligible patients or where they can form partnerships with facilities that do, e.g. assisted living facilities, nursing homes, or hospices.

Avoiding insurance entirely by ‘going cash' was one way mentioned to avoid DIR fees, but doing so raised two concerns. First, participants worried about patients forgoing services they had to pay for upfront. Second, where insurance records no longer capture the medications dispensed, cash payments could negatively affect apparent adherence rates and increase fees.

Participants believed that they needed to expand existing revenue streams. Participant 12 stated, ‘With margins as tight as they are you need to make sure you're doing everything you can. You gotta be doing stuff besides licking and sticking. You gotta be licking and pushing.' The Community Pharmacy Enhanced Services Network’s (CPESN®) USA Clinical Drug Trial Program creates opportunities for pharmacists to be involved in clinical trial recruitment. However, some participants had negative experiences with clinical trials, particularly where they did not serve enough trial-eligible patients. In 2020, the state of Missouri started to allow individual pharmacists to become individual providers. However, the services Missouri pharmacists can offer are limited to those directly related to prescription medications, e.g. medication-related telehealth and medication therapy management. On the federal level, opportunities are fewer; as Participant 1 noted, ‘Since we can't bill as a provider on Medicare Part D, it's very limited.' Branching out beyond medication-specific services was possible, e.g. offering language services or health navigator services, but doing so could require hiring specialised staff, e.g. native language speakers, or could require present staff to take up additional training, e.g. training to become community health workers.

Vaccines were an alternative service that could be reimbursed on the state or federal level. Several participants discussed vaccinating whole towns during the COVID-19 crisis, because it was their duty as community pharmacists (the overarching theme). Initially, doing so was financially beneficial, but now many vaccines are also subject to fees. Compounding for non-human animals was another frequently mentioned alternative service. Participant 5 stated that they relied on compounding for non-human animals to ‘keep the lights on,’ continuing, ‘my hope is that I can survive long enough that this thing turns around and the human side becomes profitable again.'

As the list of alternative revenue streams grows, so does the workload in community pharmacy. Participants were concerned that managing all the new services could negatively impact their ability to deliver the basics. Participant 9 states, ‘At the end of the day, the prescription still has to be filled. … Where's the time? And, we're being paid pennies to fill a prescription and manage absorbing so much time that there's no time left.'

#### Theme 3: anticipated challenges

The third theme describes anticipated challenges. Participants did not expect the policy change to increase reimbursement rates for the basics. Participant 4 predicted, ‘We will be punished, the pharmacies will be punished, I will be punished for filling.' Participant 3 described continuing workload concerns, ‘As far as I know, the star ratings are remaining the same. You still have to try to perform and do all your MTMs [medication therapy management]. You still got to perform and make those adherence calls, do the pill packs … ' Participant 6 was thankful that fee transparency would increase but remained pessimistic about reimbursement amounts, ‘The benefit for pharmacy is pharmacy at least knows how much they're losing at the point of sale on that prescription or making, but probably losing.'

Participants anticipated more pharmacy closures, decreased staffing, reduced store hours, and more central fillings. On closures, Participant 9 noted, ‘They found a way to squeeze us out. They don't have to buy us out. They just have to wait their time.' Participant 7 observed that ‘Even the chains aren’t making the money that they used to. They're cutting back on labor. … They're adding on the additional prescriptions without adding any additional labor.' All these concerns were expressed alongside concerns about diminishing patient access and decreasing safety standards (overarching theme).

A commonly expressed concern involved whether pharmacies could continue to stock medications subject to high fees. Straightforwardly, Participant 8 explains, ‘We have to dispense the medication if we have it in stock, but the law doesn't require us to dispense that prescription for the patient if we don't have it in stock.' After participants expressed that pharmacies may not stock high-fee medications, the interviewer asked where patients would get their medications. Participants quickly responded that they could use mail order. This raised safety concerns around temperature controls, delays, package theft, deliveries to the wrong address, etc. Participant 12 recounts, ‘We've heard nightmares about patients having delays on a specialty drug that's mailed out and for one reason or another it doesn't get delivered on time or it sits on the porch.’ Participant 7 notes, ‘There's no temperature controls. I mean if we had those temperatures in our pharmacies, the board of pharmacies would tell us all those drugs are bad and shut us down.'

No participants wanted to turn patients away. Expressing the purpose of pharmacy (overarching theme), Participant 10 notes, ‘We're here to serve the community. So, until such time that I really can't do it, I'm going to take care of my community. These people have been loyal to us from before they were Medicare Part D beneficiaries.' This attitude could prove a problem for pharmacy, as Participant 3 notes, ‘The pharmacy benefit managers know that independent pharmacies are not gonna let their community down. They’re not going to not take care of the patient. And, in the process, community pharmacies sacrifice themselves.'

#### Theme 4: what is needed for payment reform

The fourth theme describes what participants believe is needed to realise payment reform. Many believed that greater awareness of payment structures was needed. Regarding the broader healthcare system, Participant 4 expressed frustration over how a representative of their pharmacy benefit manager did not know what a DIR fee was. Participant 3 expressed how decreasing access to pharmacies could impact job availability and workloads for other sectors. Regarding patient awareness, participants thought that knowing the fees at the point of sale could empower more meaningful conversations. Participant 8 explains, ‘I think you will see pharmacists begin to talk more with their patients about what's going on and explain to them, you know, why they can or cannot fill certain prescriptions.'

Participants especially felt that greater awareness was needed among pharmacists. Participant 2 said, ‘Most pharmacists don't even know what a DIR Fee is.' Participant 8 agreed, saying, ‘A lot of pharmacists are just taking that and going: ‘Well, we made money over here, so we'll just pay this DIR out, and that's what it is.’’ Some participants expressed concerns that they did not learn enough about payment structures in school. Describing students, Participant 3 said, ‘They really have to get politically involved. That's the number one thing that they have to do. Unfortunately, most student pharmacists don't see it or want to be politically active.'

The current payment structures are legal. Participant 11 states, ‘The number one thing that can help with the DIR fees is legislation.’ Participants described several organisations that could combine voices and act as legislative bridges, e.g. the Community Pharmacy Enhanced Services Network, the National Community Pharmacists Association, and the Missouri Pharmacy Association. Participants expressed more positive feelings about changes at the state than at the federal level. Participant 6 stated, ‘I wish the federal government was more proactive. Usually, we wait till there's a problem. Pharmacies close. Then we try to figure out what to do. A lot of a lot of people are screaming right now that we've got a problem.'

## Discussion

Our interviews identified four main themes about how payment models impact community pharmacies’ abilities to improve public health in the places people live. First, existing payment models do not sustainably support the basic component of pharmacy practice, and so are unlikely to support further aspirations to improve patient outcomes. Second, diversifying the business model has created additional income streams but their sustainability is uncertain. Third, despite the policy change, participants anticipate growing challenges around medication accessibility and safety. Fourth, payment reform will require greater awareness of payment models. A final overarching theme stressed that the purpose of community pharmacy was to serve patients within the greater healthcare system.

As discussed in the introduction, Richard et al.’s (2021) study found that successful implementation of performance-based pharmacy payment models requires alignment of stakeholder aims, cultural shifts supporting quality improvement, and pharmacy-level financial incentives. This has not occurred. Pharmacists who participated in this study feel punished for performing the basic components of their job. They doubt the current payment models incentivize quality improvement, as even top performers experience penalty fees.

Presently, many pharmacy benefit managers do not financially value the legally required basic component of pharmacy practice: safely dispensing medications, inclusive of drug utilisation reviews and patient counselling. Hochberg (2022) provides an example of the financial value one plan attributed to dispensing Paxlovid: an oral medication to treat mild-to-moderate COVID-19 symptoms that was subject to ‘no ingredient cost' reimbursement during the pandemic. After pharmacists dispensed Paxlovid, they were charged $0.07 for submitting a claim, and the pharmacy benefit manager reimbursed $0.08. In this example, pharmacies netted only $0.01 before retrospective DIR fees were applied. The DIR policy change does not remedy this problem. The problem extends further, as the Omnibus Budget Reconciliation Act of 1990 requires drug utilisation review and patient counselling. Where pharmacies are not appropriately reimbursed for dispensing, they incur additional financial losses for time spent performing reviews or counselling patients. There is debate about what drug utilisation reviews and patient counselling should entail, but there is no recommendation to forgo such practices completely; doing so would violate ethical standards (Howell et al., 2021; Reynolds & Rupp, 2016).

Conducting good drug utilisation reviews requires access to holistic medication profiles. When pharmacists see adverse drug combinations, say between omeprazole and dasatinib (Pape et al., 2016), they could contact the prescriber to approve adjustments. Unfortunately, dispensing these medications is often fractured. While omeprazole is commonly dispensed by a local community pharmacist, dasatinib is commonly dispensed through a specialty pharmacy. This renders drug utilisation reviews less effective because these pharmacies do not have holistic medication profiles. Such fractured care is likely to increase if pharmacies reject healthcare contracts that are detrimental to their business. One could assert that pharmacists have a legal obligation to dispense all medications if appropriate for the patient, but a pharmacy that has gone bankrupt cannot serve any patients. The concept of pharmacy deserts captures how in some areas there are no accessible pharmacies. Deserts are often defined in terms of mileage, but accessibility extends to what plans are accepted, staffing, and operating hours (Ying et al., 2022). Accessibility may decrease even in areas where pharmacies remain open.

Pharmacies offer services beyond medication dispensing. Where those services benefit public health, pharmacies could be appropriately compensated. Comprehensive reviews suggest that clinical pharmacy services can be cost-effective (Talon et al., 2020; Touchette & Perez, 2023). However, nuanced decision-making is required for cost-effective analyses, including, e.g. what outcomes to assess and what services to compare. For example, research suggests that training pharmacy technicians to act as community health workers may improve community health (Yoon et al., 2024). But whether those services should compensate for money lost on medication dispensing is a complicated question. After all, each alternative service adds complexity and increases workload. Safely dispensing medications requires time to remedy problems identified during drug utilisation review (Rupp, 2020). Where that time is not built into the otherwise efficient dispensing process, safety could be compromised.

### Strengths and limitations

As the notion of information power guided our sample size, we only needed a small number of participants who were knowledgeable about the payment structures influencing their practices to identify broad themes (Malterud et al., 2016). That said, recruiting even a small number of such participants was challenging. This reiterates our findings that greater awareness is needed. Where the financial side of a practice is outsourced, even owners could be unaware of costs. Further, note that only 1 of our 12 participants identified as female. This gender disparity could reflect female pharmacists choosing not to engage in the financial side of their practice (Eiland et al., 2022). Regardless of whether a pharmacist chooses to lead the financial side of their practice, it is advisable to understand the payment models.

The present study brings together the voices of those affected by current policies to shape future research and inform policy reform. The present study does not test a particular hypothesis but could serve as the foundation for future quantitative investigations, e.g. around medication availability or mail-order safety. The present research focused on participants in a single state, Missouri. Opportunities for pharmacies to develop alternative services differ across states, and how state policies fit together is complicated. Medicare Part D is a federal programme, and changes in Medicare policies impact all states. Thus, changes at a federal level could more efficiently improve public health. A larger study could use a stratified recruitment method across states to capture how federal policies affect state policies. If sufficient resources are available, such research could be conducted following a participatory research framework that brings together pharmacists, patients, and policymakers to ultimately improve public health (Cornwall & Jewkes, 1995).

## Conclusion

Community pharmacies require a financially viable and sustainable business model to deliver the basic component of practice: safely dispensing prescription medications. Our interviews with community pharmacists suggest that the present payment models are insufficient. Pharmacists feel as though they are being punished for performing their basic functions and do not anticipate that the DIR policy change will improve their sustainability. Even as pharmacists increase the amounts and types of services offered, without payment reform, they believe that pharmacy services will become less accessible. To realise payment reform, particularly on the federal level, greater awareness of existing payment models is needed.

## Supplementary Material

Supplemental Appendices

## Data Availability

The transcript data are not publicly available due to restrictions, i.e. their containing information that could compromise the privacy of research participants. Representative quotes of the themes and subthemes derived from our analysis are available in the Supplemental Appendices online.
