# Supplementary material for: Payment models and the sustainability of community pharmacy practice: a qualitative interview study with community pharmacists
Source: J Pharm Policy Pract. 2025 Jan 13;18(1):2450018. doi: 10.1080/20523211.2025.2450018 (PMC11730775; doi:10.1080/20523211.2025.2450018)
Supplement: Supplemental Appendices [file JPPP_A_2450018_SM6141.zip › Supplemental material/Supplemental Material.docx]

**Supplemental Material**

**Appendix 1. COREQ Checklist**

Consolidated criteria for reporting qualitative studies (COREQ): 32-item checklist

| **No** | **Item** | **Guide questions/description** |
| --- | --- | --- |
| **Domain 1: Research team and reflexivity** | | |
| Personal Characteristics |  |  |
| 1. | Interviewer/facilitator | Which author/s conducted the interview or focus group?  KS (the primary investigator) conducted the interviews. This is specified in the “Methods – Data Collection” section. |
| 2. | Credentials | What were the researcher's credentials? *E.g. PhD, MD*  KS has a PhD. This is specified in the “Methods – Data Collection” section. The content of the interview guides were informed by a second researcher, i.e., TW (RPh, FAPhA; PhD; Female). |
| 3. | Occupation | What was their occupation at the time of the study?  KS was an assistant professor of psychology at the time of the study. This is specified in the “Methods – Data Collection” section.TW was employed by a college of pharmacy at the time of this study. |
| 4. | Gender | Was the researcher male or female?  KS and TW identify as female. This is specified in the “Methods – Data Collection” section. |
| 5. | Experience and training | What experience or training did the researcher have?  KS has training in mixed-methods techniques and over 10 years of experience conducting research in applied health contexts. This is specified in the “Methods – Data Collection” section. TW has conducted and published qualitative and quantitative research in pharmaceutical practice. |
| Relationship with participants |  |  |
| 6. | Relationship established | Was a relationship established prior to study commencement?  KS had no prior relationship with the participants prior to the study commencement. This is specified in the “Sample Size/Participant Selection” section |
| 7. | Participant knowledge of the interviewer | What did the participants know about the researcher? e*.g. personal goals, reasons for doing the research*  All participants provided their informed consent before participating. The information sheet explained the purpose and nature of the study, that participation was voluntary, and that identifiable data would be redacted from reports. This is specified in the “Ethical Approval” section. |
| 8. | Interviewer characteristics | What characteristics were reported about the interviewer/facilitator? e.g. *Bias, assumptions, reasons and interests in the research topic*  Characteristics of the interviewer are described in the “data collection” section. |
| **Domain 2: study design** | | |
| Theoretical framework |  |  |
| 9. | Methodological orientation and Theory | What methodological orientation was stated to underpin the study? *e.g. grounded theory, discourse analysis, ethnography, phenomenology, content analysis*  The methodological approach was pragmatic; no prior framework or theory was assumed before data collection commenced. This is specified in the “Study design” section. |
| Participant selection |  |  |
| 10. | Sampling | How were participants selected? *e.g. purposive, convenience, consecutive, snowball*  Participants were purposely selected and then further recruitment was carried out using snowball techniques. This is specified in the “Sample Size / Participant Selection” section. |
| 11. | Method of approach | How were participants approached? e*.g. face-to-face, telephone, mail, email*  Participants were approached via email by our research team. This is specified in the “Ethical Approval” section. |
| 12. | Sample size | How many participants were in the study?  Twelve participants were in the study. This is specified in the “Sample Size/Participants Selection” section. |
| 13. | Non-participation | How many people refused to participate or dropped out? Reasons?  Five participants gave reasons for not taking part. This is specified in the “Sample Size/Participants Selection” section. |
| Setting |  |  |
| 14. | Setting of data collection | Where was the data collected? e*.g. home, clinic, workplace*  Via a recorded Microsoft Teams or telephone call. This is specified in the “Data Collection” section. |
| 15. | Presence of non-participants | Was anyone else present besides the participants and researchers?  Interviews were conducted where participants felt comfortable. For some participants this was in a private location, whereas for other it took place in a shared office. This is specified in the “Data Collection” section. |
| 16. | Description of sample | What are the important characteristics of the sample? *e.g. demographic data, date*  This is specified in the “Participant Characteristics and Demographics” section, along with in Table 1. |
| Data collection |  |  |
| 17. | Interview guide | Were questions, prompts, guides provided by the authors? Was it pilot tested?  The semi-structured interview guide was pilot tested with to professors of pharmacy. This is specified in the “Study Design” section.  The interview guide is provided in Appendixes. |
| 18. | Repeat interviews | Were repeat interviews carried out? If yes, how many?  A single interview was carried out with each participant. This is specified in the “Data Collection” section. |
| 19. | Audio/visual recording | Did the research use audio or visual recording to collect the data?  Yes, i.e., a recorded Microsoft Teams or telephone call. This is specified in the “Data Collection” section. |
| 20. | Field notes | Were field notes made during and/or after the interview or focus group?  Yes, field notes were recorded during the interviews. Brief summaries of the interview were composed directly after each interview took place. This is specified in the “Data Collection” section. |
| 21. | Duration | What was the duration of the interviews or focus group?  About 30 minutes. This is specified in the “Data Collection” section. |
| 22. | Data saturation | Was data saturation discussed?  No. The research team agreed that “THEMATIC saturation was reached after 9 interviews. This is specified in the “Data Analysis” section. |
| 23. | Transcripts returned | Were transcripts returned to participants for comment and/or correction?  No. The research team agreed that “THEMATIC saturation was reached after 9 interviews. This is specified in the “Data Analysis” section. |
| **Domain 3: analysis and findings** | | |
| Data analysis |  |  |
| 24. | Number of data coders | How many data coders coded the data?  Five researchers were involved in coding and theme development. This is specified in the “Data Analysis” section. |
| 25. | Description of the coding tree | Did authors provide a description of the coding tree?  A concept map is provided in Figure 1 and in supplemental materials. |
| 26. | Derivation of themes | Were themes identified in advance or derived from the data?  Themes were derived from the data. This is specified in the “Study design” section. |
| 27. | Software | What software, if applicable, was used to manage the data?  Microsoft Teams was used to collect data. This is specified in the “Data Collection” section.  Code and theme development was conducted using Microsoft Word’s comments function, Excel to list codes, NVIVO v14 to identify direct quotes, and PowerPoint to arrange themes into a concept map with hierarchical levels. This is specified in the “Data Analysis” section. |
| 28. | Participant checking | Did participants provide feedback on the findings?  Participants were sent a draft of the manuscript to provide feedback. This is specified in the “Data Analysis” section. |
| Reporting |  |  |
| 29. | Quotations presented | Were participant quotations presented to illustrate the themes / findings? Was each quotation identified? e*.g. participant number*  Yes, quotes are provided to illustrate themes. Participants are identified via a number. This is provided in the “Results” section. |
| 30. | Data and findings consistent | Was there consistency between the data presented and the findings?  The unit of analyses was the theme rather than the prevalence or frequency of statements. Some statements of quantification are included (e.g., statements such as often and rarely), but do not always aim at providing estimates of prevalence outside the present participant sample. |
| 31. | Clarity of major themes | Were major themes clearly presented in the findings?  Yes, this is provided in the “Results” section. |
| 32. | Clarity of minor themes | Is there a description of diverse cases or discussion of minor themes?  Yes, this is provided in the “Results” section. |

**Appendix 2. Semi-structured Interview Guide**

**INTRODUCTION – CONSENT AND RIGHTS TO WITHDRAW**

Researcher: Thank you for agreeing to take part in this interview about direct and indirect remuneration fees to help us understand your perspective as a community pharmacist. This interview may last 30 minutes and will be recorded. Before we get started, can you please confirm that you have read the information sheet and provided your informed consent to participate in the study titled: A qualitative study to understand the impact of direct and indirect remuneration (DIR) fees on community pharmacies in Missouri from the perspective of community pharmacists.

- Participant [if yes, the interview continues, if no, the interview ends; the participant may be asked to provide their informed consent and to reschedule the interview]

Researcher: Thank you. Now, I want to remind you that you have the right to withdraw at any time during the interview and up to two weeks after this interview is complete or after you have had the opportunity to review your transcript. Do you have any questions before the interview starts?

- Participant: [either asks questions to which the researcher responds or does not have any questions].

Researcher: Great. Could you describe if anyone else is in the room with you and what their job titles are?

- Participant: [either describes or says no one else is there].

Researcher: Thank you. Moving forward in the interview I am going to ask you some structured questions about yourself, your pharmacy, operating costs, and reimbursement fees. Then the interview should become more flexible and guided by what you feel is most important.

**Participant/Pharmacy Characteristics**

*(only need to ask if participant did not already answer on Calendly – If already answered just confirm answers here --[I’ve seen you’ve already answered many of the structured items. According to my records your job title is [state] and you’ve worked in your current practice for [number] years. You estimated that your pharmacy serves [number] patients annually, that you have [number] full-time staff and that there are approximately [number] other pharmacies within a 10-mile radius of your practice. Is this all correct? [if yes, move on, if no revise].)*

Researcher: There are four questions about you and your pharmacy characteristics. First, please describe your job title and how many years you have worked with your current pharmacy.

- Participant: [says job title and years in practice]

Researcher: Please estimate the number of patients your pharmacy serves annually.

- Participant: [number]

Researcher: Thank you. How many full-time staff does your pharmacy currently employ?

- Participant: [number]

Researcher: Approximately, how many other pharmacies exist within a 10-mile radius?

Participant: [number]

**Operating and DIR costs**

*(only need to ask if participant did not already answer on Calendly – If already answered just confirm answers here [I’ve seen you’ve already answered many of the operating and DIR items for us. According to my records your annual operating budget without DIR fees is approximately [dollars] annually, your DIR fees in 2022 were [dollars] and you anticipate they will be [dollars] in 2023. Is this correct? [if yes, move on, if no revise].)*

Researcher: Great, Now I have four quick questions about your pharmacy’s operating costs and direct and indirect reimbursement fees. First, how much is your annual operating without DIR fees budget?

- Participant: [operating budget]

Researcher: What percent of your business is in Medicare Part D?

- Participant: [percentage]

Researcher: Okay, second question: How much were your DIR fees in 2022?

- Participant: [DIR fees in 2022]

Researcher: Thank you, and this is the last question: what are your projected DIR fees in 2023?

- Participant: [DIR fees in 2023]

**Open-Ended Semi-Structured Questions**

Researcher: Great, the structured questions are done. Now the interview should become more flexibly guided by your perspectives. I may ask your questions to be sure I understand and capture this perspective.

First, please describe your understanding of DIR as it pertains to pharmacy practice.

- Participant: [speak freely]

Follow-up/probe questions to be used flexibly:

- - In your opinion, what factors contribute to the variability of DIR fees across different insurance plans and pharmacy networks?
  - Can you share any challenges or frustrations you have encountered when it comes to calculating and reporting DIR fees?
  - Do you contract with a pharmacy services administrative organization (PSAO), and why?
  - Researcher: Are there any aspects of the new DIR policy that you feel are unclear or need further clarification? If so, what are they and how do you think they should be addressed?

Researcher: What services beyond medication dispensing could you offer that you could use to recoup lost revenue?

- Participant: [speak freely]

Follow-up/probe questions to be used flexibly:

- - How are these services reimbursed?
  - Can your patients’ needs be met via telehealth systems?

Researcher: In your opinion, what are the potential benefits or drawbacks of this new policy for patients, pharmacies, and other stakeholders? You may choose which group you start.

- Participant: [speak freely]

Follow-up/probe questions to be used flexibly:

How might your practice be impacted?

- - Do you think your practice will be affected differently than other practices? And Why?

How might your staff be impacted?

- - How might this affect their families?
  - What are other employment opportunities for staff in your area?
  - How might your patients be impacted?
  - Can your patients’ medications be mailed?

Researcher: Given that DIR fees are closely linked with Medicare Part D, is it better to not provide medications for part D beneficiaries and why?

Researcher: What support or resources do you think would be helpful for pharmacies in adapting to and complying with the new DIR policy?

**Closing questions**

Researcher: Thank you. I have just a few more quick questions before we end the meeting. First, would you like to review a copy of your transcripts before we include them in the analysis?

- Participant: [If they would, then after transcripts are produced these participants will be sent a copy and given 2 weeks to assess the transcript’s accuracy and recommend modifications.]

Researcher: Second, would you like to receive a copy of our final report?

- Participant: [If they would, then we will request permission to save their email address in a separate file from their research data, without any linking codes.]

Researcher: It has been great to speak with you today. Do you have any other questions, comments, or concerns you would like to express before we end this interview?

- Participant: [either offers questions to which the researcher responds or does not have any questions].

Researcher: If we need to your help contacting other pharmacists to take part in this research, would you be happy to introduce us?

- Participant: [if yes, say that they may be recontacted if no then record and do not recontact them]

Researcher: Thank you for taking the time to speak with me today. Please feel free to contact me at the email address provided in the information sheet if you have any additional questions.

**Appendix 3. Concept map with additional representative quotes for each theme**

Attached as a separate electronic PPT file.
