# Supplementary material for: Payment models and the sustainability of community pharmacy practice: a qualitative interview study with community pharmacists
Source: J Pharm Policy Pract. 2025 Jan 13;18(1):2450018. doi: 10.1080/20523211.2025.2450018 (PMC11730775; doi:10.1080/20523211.2025.2450018)
Supplement: Supplemental Appendices [file Appendix3.pdf › Supplemental material/Appendix3.pdf]

(Themes and representative quotes)

Need for Payment Reform in Pharmacy Practice

Theme 1: Being punished for the "basics"

Increasing Fees

P3 - That's how much it [DIR fees] increased from 2010 to 2019. 91,500%. So that's equivalent to a four-dollar gallon of milk going up to $3,660.00 in the span of nine years.

P3 - It [DIR fee] progresses, getting worse year after year, even over the past six years, it just doesn't go any lower.

P2 - I think in the last 10 years, it's gone up 107,000%, the DIR fees. So that huge number that I'm hearing from NCPA, which is the National and Community Pharmacy Association.

P12 - It's just been getting worse and worse because the DIR percentage has gotten bigger and bigger.

P11 - There's some statistic that the fees have increased 110,000% in ten years. There's no good reason that I can think of for why anything would increase that much in that amount of time. … The rate of increase has just been astronomical. It is not easy for any business to deal with.

P3 – Where is that money going? I mean, if it were improving quality care, OK. But I feel like that's not the case. It's still the same quality care. We have been providing our patients with five-star quality care ever since our inception.

P7 - DIR fees are basically supposed to help motivate us to motivate the patient to take their medications. That's the whole purpose of them. But, over the years the DIR fees get bigger and bigger, even though the reimbursement has not gotten better. But the net effect was no, not yet.

P12 - The last couple of years the hill, the up and downs on cash flow and independent pharmacy, it's just been getting worse and worse because the DIR percentage has gotten bigger and bigger.

P6 -The metrics that are applied to DIR calculations are applied at a health plan level, not at the pharmacy level, but a health plan level. So really, it's metrics that are played by Humana, by Edna, by United Healthcare. It's at their level. It's claims that go to their plan. And, the problem is that once that gets down to a pharmacy level.
-- I'm sure you've done plenty of research. In order to have a valid feasible reliable measure, you have to have a certain level of validity from the denominator. So, it has to be, it can be reliable measure, right? If you have a denominator of say 5, you don't have a reliable measure, because your denominator is not big enough for the population. ---

Low reimbursements for the basics

P5 - … for customers that come in for a depo testosterone shot, I know the minute they walk in the door I literally give them $10 out of my pocket because they're paying me a price lower than I can purchase it for and they know it. Because I can't really dispense a 10 mil vial.

P8 - I had a patient come over from [a big chain] the other day. They had a $40 copay. That was the total reimbursement. The PBM [illegible] paying no money. They just contracted it out to a $40 co-pay and it was about that cost on three different meds. They came over [illegible] they had to pay me was only like $7.00. Nobody's even making money there. They're [PBMs are] just trying to push me out of the [illegible] these guys are predators.

P10 - This goes beyond DIR fees because on many plans like commercial plans plan, you [patients] come in for a weig[illegible] hot topic right now. People probably think that pharmacies are cleaning up. While they're not. …The reimbursemen[illegible] what I paid for it.

P5 - I learned to live with low profit margins. But when you're at 6.1% of sales, in your overall profit over costs, not profit over expenses, profit over cost is 15 to 16%. You can't win.

P7 - It's down to the reimbursement. Even the chains aren't effective. They're not making the money that they used to.

P5 - A customer that came in today and had eight prescriptions, and I was literally losing [illegible] The minute they walk in, every month, I know I give them 1000 dollars out of my out of [illegible] can't turn away that customer away. I've been taking care of that customer since [illegible]

P5 - We're being paid pennies to fill a prescription. In some cases we're being paid negative pennies to fill prescriptions, while in a lot of cases. That's gonna stop, yeah.

P5 - It's possible to run the pharmacy at 17% and 19% over cost but wh[illegible] 16% you don't have room for growth you don't have room for anythi[illegible] being asked to do right now. It's because of the shena[illegible]

P5 - At the profit margin we're making, where is the money to finance the next machine because it's empty, and I'm gonna have to do it because of safety. But where's the profit margin? It's not there.

P5 - Where am I going to get the money to update my automation and w[illegible] store the profit margin is not there anymore. And why is the profit margi[illegible] the Medicare DIR fees. Who would have ever thought that last year th[illegible] worth of Medicare DIR fees and it's not accountable. And that's [illegible]

P3 - The problem is that the reimbursement of these is just so bad that I mean, I wanna say our net profits on last year was like $1.27 per prescription on all Part D plans put together. We were likely profitable on maybe two of the Part D plans and on other ones we actually lost thousands of dollars due to the DIR fees. Before the DIR fees we were fine. But after the DIR taken away, then we were very negative on a lot of them.

P10 - If the drug cost $50[illegible] say, $550 and then my D[illegible] $10 and that's that is 100[illegible] name drugs, we ultimat[illegible]

P12 – They would probably be sending them to a chain pharmacy down the street where the pharmacist has no idea what th[illegible] brand-name drug. They're just filling the prescription and getting out the door as fast as they can. They have no idea. They're [illegible] That's part of it. They're based on how many prescriptions they check in a given hour. They're constantly being scruti[illegible]

P10 - When you look at the reimbursements either on generic drugs or brand name drugs, they are very, very, very small. Th[illegible]

P5 - [name of pharmacist who went out of business] took care of the town. I took care of her town. She was a pharmacist of [illegible] I'm 30% busier. That pharmacy should not have disappeared. But I was old enough to see what was going on. She was brand [illegible] low paying insurance realities. I absorbed 100 prescriptions. And that was October 2022 when she folded. I'm still trying to fi[illegible] back from my wholesaler because I'm so brand intensive. At one point I was offshoring 160,000 hours with a brand a month t[illegible] my rebate back for my generic was not significant because my brand was branded a sense of belonging and maturity as I'm stil[illegible] the bills even though I'm 30% busier. It's nuts. And, and the only people that are winning a CVS and Walgreens because we're [illegible] them.

P1 - At this point, it's bett[illegible]

[illegible]

Brands

[illegible]

How performance is measured

[illegible]

Lack of Transparency

[illegible]

Theme 2: Diversifying the Business Model

Beyond the basics

[illegible]

Ways to fill

[illegible]

Sources

[illegible]

Alternative services to offer

[illegible]

Contracting

[illegible]

Problems getting paid

[illegible]

Shouldn't need to subsidize the "basics"

[illegible]

Theme 3: Anticipated Challenges with Policy Change

Pessimistic about change

[illegible]

Pharmacy Closures

[illegible]

Decreasing Access

[illegible]

deserts

[illegible]

Staffing/ hours / Central Filling

[illegible]

Stocking

[illegible]

Mail Order

[illegible]

Theme 4: What is needed for Payment Reform?

Organizations Combining Voices

[illegible]

Healthcare system

[illegible]

Awareness

[illegible]

Patients

[illegible]

Pharmacists

[illegible]

Legislation

[illegible]

Cross-Cutting Theme: Purpose

Who are community pharmacists?

[illegible]

Patients/communities

[illegible]

Who do community pharmacies serve?

[illegible]

Health Care System

[illegible]

Who do PBM's Serve

[illegible]
